# Moving Beyond “Check A Box”: Shifting Physician Perceptions and Culture with an Antiracism and Equity Curriculum

**DOI:** 10.5811/westjem.20797

**Published:** 2025-05-19

**Authors:** Hannah Barber Doucet, Timmy Lin, Taneisha Wilson

**Affiliations:** * Boston University Chobanian & Avedisian School of Medicine, Department of Pediatrics, Boston, Massachusetts; † Alpert Medical School of Brown University, Department of Emergency Medicine, Providence, Rhode Island

## Abstract

**Objectives:**

The purpose of this study was to evaluate the impact of the Discussing Anti-Racism and Equity (DARE) curriculum on individual physician knowledge and practice, as well as on perceptions of group culture.

**Methods:**

DARE was a longitudinal multimodal curriculum targeted at pediatric and adult emergency medicine (EM) trainees and faculty, made up of 12 lectures/workshops, three simulations, five book clubs, and two movie screenings. We used a multiphase, parallel convergent mixed-methods approach. Focus groups before and after DARE explored prior education, antiracism attitudes and behaviors, perceived impact of intervention curriculum, and perceptions of departmental medical culture. We elucidated themes using thematic analysis. Surveys of trainees and attendings evaluated individual attitudes and practices related to equity and antiracism.

**Results:**

We held nine focus groups with a total of 52 participants. Half of participants were EM residents (26), and half were faculty (12 pediatric EM and 14 general EM). Four major themes emerged around antiracism education and DARE. Both trainees and faculty reported a lack of standardized or effective prior education, although trainees are beginning to report increased exposure in medical school. Participants reported an overall positive impact of DARE on individual knowledge and practice, with continued room for improvement. Focus groups particularly highlighted a perceived shift in departmental antiracist culture post-DARE. Finally, future curricular aims were elucidated. A total of 56 surveys showed significant improvement in all realms of antiracism medical- practice questions when posed as retrospective pre-post questions (P < 0.01).

**Conclusion:**

The DARE curriculum increased individual antiracism awareness and cultivated culture shift among the targeted clinician group. Focus groups provided clear next steps for ongoing and expanded education.

## BACKGROUND

Health inequities stemming from systemic, interpersonal, and internalized racism have plagued our medical system for as long as it has existed.^1^ The medical community at large, from physicians to hospitals to journal editors, has issued calls to address health inequity and structural racism in medicine.^2^–^4^ A recent review of the emergency medicine (EM) literature showed the persistence of racial inequity in our field, including in pain treatment, stroke management, timely or appropriate antibiotic therapy, rates of physical restraint, and survival rates following out-of-hospital arrest, to name just a few.^5^ Racism does not skip over children either; racial disparities impacting pediatric emergency department (ED) care include pain treatment,^6^ physical or pharmacological restraint use,^7^,^8^ sepsis morality rates,^9^ and evaluations for child abuse.^10^ Our field needs a multipronged approach to create an antiracist practice of medicine and alter inequity.^11^–^13^ Impactful education will be one key driver in that change.^14^,^15^

A variety of educational interventions have been proposed to address racism in medicine, including many one-time workshops and some longer curricula (typically single format).^16^–^18^ However, it remains unclear how to best impact individual racism/implicit bias, and few interventions have assessed educational impact on a broader shared culture of medicine. Considering this, we piloted a curriculum called Discussing Anti-Racism and Equity (DARE) for emergency and pediatric emergency clinicians. This comprehensive year-long educational intervention was designed to increase antiracist attitudes and behaviors among emergency clinicians, with a broader goal of improving patient care and shifting departmental culture. The DARE curriculum consisted of a variety of educational modalities including book clubs, simulation sessions and workshops, lectures, movie screenings, and targeted clinical cases during morbidity and mortality conferences. We used a mixed-methods assessment to evaluate the impact of the intervention, focusing on antiracist attitudes and behaviors as well as impressions of departmental culture.

## OBJECTIVES

The objective of the DARE curriculum, broadly, was to encourage anti-racist attitudes and behaviors among emergency clinicians. Specifically, participants would be able to do the following:

1. Assess implicit bias and the effects of racism on their individual practice of medicine.
2. Identify the legacy and impact of structural racism on medicine.
3. Use practices that challenge the subtle impacts of individual racism/bias in their clinical work and interpersonal interactions.
4. Take actions or create feasible goals toward an actively anti-racist practice of medicine.
5. Participate effectively in an actively anti-racist departmental culture.

The curriculum had an additional objective to develop a core group of participants committed to developing and demonstrating an anti-racist ethic in our ED.

## CURRICULAR DESIGN

We recognized the need for an anti-racism and equity-focused curriculum as there was little to no formal training in these topics at our institution. Both our faculty and trainee group were majority White, as was our nursing staff. The population our hospitals served was more diverse. The curriculum and its evaluation included physicians (attendings, fellows or residents) from three hospitals, comprising one pediatric ED and three general EDs. Our largest hospital, including a pediatric and adult ED, averaged seeing at least one-third Hispanic- or Black-identifying patients.

We were aware from our own experiences (as a Black female emergency attending and White queer female pediatric EM fellow) as well as those shared with us by others that examples of microaggressions and potentially biased patient care were common. Contextually, the DARE curriculum started in the fall of 2020, directly following the murder of George Floyd and the subsequent growth in general anti-racist awareness as well as political backlash. This meant that many in our faculty group were more invested in the idea of the DARE curriculum and perhaps were primed to embrace a cultural shift. However, for a politically divided ED staff, it also meant significant new obstacles in engaging nursing and hospital staff, as well as an urgent need to help our trainees navigate and have support around potentially fraught interactions within the ED.

In building the DARE curriculum, we used the levels of racism as outlined by Camara Jones,^19^ as well as Bloom’s taxonomy,^20^ to ensure that it was of appropriate scope and depth (Table 1).

To encourage knowledge retention and skill growth, sessions occurred longitudinally and built on each other, as well as allowed participants to practice previously learned skills. To target our learning objectives within our specific hospital and departmental context, initial sessions focused on White identity and privilege, microaggressions and implicit bias, as well as introduction to racism and medical racism. After introductory sessions we integrated skill-building practice, with three simulation sessions and a case-based workshop scattered through the year. Ongoing lectures continued to grow participant knowledge base. All of these sessions were held during scheduled, protected conference time, allowing a majority of trainees to attend; faculty were strongly encouraged to attend. We additionally worked with our department to fold antiracism content into other available learning opportunities. Notable examples include a morbidity and mortality session focused on racism in real patient cases, run by EM chief residents, and requesting that the grand rounds committee invite a topical speaker.

To address different learning styles and allow for more in-depth learning, we also used a book club model (with books provided) and movie screening with discussion. Due to time constraints, these sessions were held outside educational conference time and had correspondingly lower attendance. However, they allowed participants time for more robust conversation and relationship-building and assisted in our final curriculum goal of growing out a core of participants who could be counted upon to be more active ambassadors and advocates in our department. The first year of the curriculum had a total of 12 conference sessions, three simulation scenarios,^21^ five book clubs, and two movie screenings with discussions (Table 2).

## IMPACT/EFFECTIVENESS

### Methods

We evaluated the curriculum using a multiphase, mixed-methods study with a parallel convergent approach. In the initial phase, focus groups at the start of the curriculum focused on an educational needs assessment and baseline understandings of ED culture around race and racism. In the second phase, focus groups held mid- and post-curriculum focused on an assessment of the curriculum, including any perceived changes to group understandings of culture. Focus groups were chosen for this methodology to examine shared social meanings and meaning-making in how race and racial bias are viewed by emergency physicians, and how this coalesces into a broader local medical culture. Interactive consensus was important to such a process. The quantitative portion was a web-based anonymous survey given post-curriculum. The survey collected information on demographics and anti-racist attitudes and behaviors. The study was deemed exempt by the Lifespan Institutional Review Board. This curriculum and study received funding via the Emergency Medicine Department of Equity Initiatives discretionary funding for faculty and resident development.

### Qualitative Approach

We created separate focus groups for residents, EM attendings, and pediatric EM attendings. We considered dividing groups further by race/ethnicity; however, given the small number of non-White physicians in our department this was not feasible. To recruit as wide a sample as possible for the focus groups by making attendance easy, we held multiple resident focus-group sessions concurrently at the end of resident conference time. A simple, anonymous opt-out mechanism was in place to ensure no trainees felt pressured into participation. For attendings, we recruited for focus groups via email to all eligible participants. Groups had 4–9 participants each, with multiple focus groups recruited within each subgroup. Given the timing of focus groups during the COVID-19 pandemic, all focus groups were held over Zoom (Zoom Video Communications, Inc, San Jose, CA). Participants received a $50 gift card after completion of the group.

Two curriculum leads (HBD, TW) developed the interview guide, based on medical implicit bias and medical education literature. Focus-group questions focused on how race and racism were discussed and perceived in the ED, to elicit the departmental culture. Other questions focused on prior education related to race and racism in medicine, and relative effectiveness of varying learning techniques. Finally, questions during and after the DARE curriculum elicited feedback on those sessions and reflections on how personal practice, patient care, and/or departmental culture may have shifted during the training sessions.

The majority of the focus groups were facilitated by a creator of the DARE curriculum. This facilitator was a White pediatric EM fellow, with a background in equity and bias-focused medical education and prior experience facilitating conversations about race and racism among medical professionals. Two additional facilitators were recruited who had prior experience in facilitation and bias work, who were both White and EM attendings. Focus groups were audio recorded via Zoom and a back-up device. Recordings were transcribed verbatim and de-identified prior to analysis.

Transcripts were organized using NVivo v12.6 (QSR International, Burlington, MA). Data were inductively coded without a priori codes to meet an exploratory goal of describing ED culture and educational preferences. A number of transcripts were double-coded until a final codebook was established. Codes were then clustered into descriptive themes using thematic analysis. Once final themes were identified, representative quotes were selected.

### Quantitative Approach

An anonymous survey was emailed to all eligible participants six months after the one-year curriculum completed. The survey included questions focused on the learning objectives of the curriculum (see Table 1), using retrospective pre/post questions referred to here as Anti-Racism Medical Practice (Appendix A). This retrospective pre/post approach was chosen as learning related to racism and implicit bias is potentially susceptible to response-shift bias.^22^ That is, participants may overestimate their understanding of issues of racism and bias, and/or underestimate their own bias, prior to educational interventions. Participants selected scaled responses that mimicked a stages-of-change model, with increasing numbers of 1 to 5 indicating increased engagement with the queried skill: 1) I am not interested in this practice; 2) I would like to do this but have not considered how; 3) I have been thinking about how to do this; 4) I sometimes do this; 5) I often or always do this. This scale was chosen based on the experience of the antiracism educators as well as findings in early focus groups that participants tended to reflect on their individual journeys in roughly such a fashion (data not reported here). Of the nine questions in this series, the first six were tabulated into a score for analysis. As the surveys were collected anonymously, survey results were not individually linked to qualitative data.

We conducted statistical analysis using SAS v 9.4 (SAS Institute, Inc, Cary, NC). Independent sample *t*-tests were conducted to compare changes in the Anti-Racism Medical Practice scores over time by several characteristics. We conducted repeated measure analysis of variance to assess the change in Anti-Racism Medical Practice scores over time by sex and race.

### Qualitative Results

Focus groups were held until we reached thematic saturation, ie, until no new major themes emerged. In total, this required nine focus groups with 52 participants. This included 12 pediatric EM attendings, 14 general EM attendings, and 26 EM residents. A variety of themes emerged in analysis, which were consistent across the adult and pediatric groups. Here, we focus on four overarching themes: incomplete prior antiracism/equity education; impact of DARE on individual knowledge and practice; departmental culture before and after DARE; and desires for the future. Each of these four overarching themes had corresponding subthemes, discussed further below and summarized in Table 3.

#### Needs Assessment: Prior Experiences and Expectations of Anti-Racist Education

Residents as well as attendings who reflected on medical school found their prior education to be inadequate or “bad.” Cultural competency content ranged from information about traditional health remedies to “ridiculous” and “bizarre” charts characterizing different ethnic groups. Some participants noted they received education about health disparities “maybe one day a week for a few weeks…at a really basic level. Pretty painful to sit through.” “Anything related to race and healthcare was kinda couched in health disparities.” However, it never “discussed how race is not biological and it did not unpack the concept of racism; it just talked about how racial minorities are more at risk for health disparities.” All participants noted any formal medical school education that touched on race or diverse populations seemed more to “check a box;” something that “we had to go through, and then we moved on.”

For some residents, experiences such as hospital rotations were more impactful than formal curricula.

Just every single patient interaction, I genuinely feel like every attending, every resident, every social worker, every interpreter, community health worker, [was a] patient advocate. It was just like the system just was different - a public hospital, that was for-the-people-by-the-people feeling. So while we didn’t get a formal education, I felt like we were taught so much every day.

Another resident noted negative lessons that could come from this non-formal learning. At her school,

the patient population is majority African-American … but we didn’t get a lot of teaching about it. And that being said … it’s kinda a joke within the school that every single chair of every department is an old white male.

Attending groups also noted the absence of structured racism-related education in prior training, recalling “things like social determinants of health” and “a social medicine focus … not in a formal way, but it was something that was out there and the mission of emergency medicine.” Or participants noted a complete absence: “I don’t remember having any conversations about race.” In this void, non-formal learning often came up as formative. Like the residents, for some this was positive.

Other attendings recalled more stereotyped experiences as trainees, such as witnessing an attending discounting the complaints of a Spanish-speaking patient with “‘That’s just status Hispanicus.’ It didn’t even register to me … that was woven into the fabric of training.” Many attendings pointed to learning outside the medical environment as their main exposure to learning about racism and related topics, in undergraduate or postgraduate programs, through experiences living in diverse areas or having cross-racial friendships or family members (for White participants), or via self-directed learning.

A final subtheme noted in the attending groups around education was the process of unlearning.

I think about all the things that we have to unlearn that we’ve been taught throughout time, throughout all of our individual lives, that you have to unlearn and unpack in a high‐speed, high‐octane situation like the emergency department. It’s really a lot.

Similarly:

How do you unfinetune some of those biases that are hurtful or harmful? We act on all of those assumptions, and it’s really hard to … recognize them when you’ve spent your whole career internalizing and acting on them.

Of note, among post-DARE groups with newer residents, the reflection on medical school content had begun to shift. Some residents specifically noted the use of taking implicit association tests (IAT) in medical school. One resident, on discovering their implicit racial bias, found it to be “a really shocking thing … a really eye-opening moment.” Another found their IAT results to be “really painful to hear” but “a really good tool.” Others commented on the changing landscape in medical schools, explaining that “especially younger physicians, I feel like bias training has been part of my educational process since the very beginning.”

#### Impact of DARE on the Individual

A major theme post-DARE was changing awareness and knowledge. Participants across stages of training noted their “recognition” that their thinking had “evolved,” or that they were “not as oblivious as I once was.” Some also referenced specific areas of learning, such as around medical mistrust, pain treatment, restraint use, microaggressions, and how bias can manifest in medical care communication. Attendings in particular reflected on teaching and leadership, finding that DARE “made me think I oversee so many learners … what I say and how I say it and how I act is really impacting the upbringing and the future careers of the people I’m around.” Attendings noticed changes in how they educated trainees and addressed team members. Some voiced new concerns in committee meetings or considered reshaping curricula.

Regarding direct patient care, many noted general awareness, whether it’s “just sitting in the back of your mind” in a variety of scenarios, or “a continual reminder of…you have to check yourself.” Some participants noted it made them change practice, such as considering race as a factor when noticing a “discrepancy” in patient care, or pausing to consider for an agitated patient “if this person was a different color … would we be sedating ’em now? Or would we be verbally deescalating now? Would we be calling family?” The ability to confront biased language was a frequently cited skill, particularly after opportunities to practice and discuss this skill in simulation. A small number had difficulty pinpointing any changes in their practice, or more commonly, reflected on their room for improvement, feeling “I’m still not 100 percent prepared,” or noting a change in thought process but “is it translating into actions yet? I’m not sure, but I’m hoping that it will.”

#### Impact of DARE on Departmental Culture

Pre-DARE residents had a negative reflection on departmental-level culture. They reflected on their inability to alter departmental practices, and brought up examples of how residents who had attempted to create change either met or worried about leadership resistance:

Just reflecting on some of the social movement events that were happening earlier this year. A lot of our mentality was like, ‘I hope we don’t get in trouble by the system.’ Not like, ‘I hope the system organizes this event so that we can all participate in it.’ It’s like, ‘No, the residents are planning, and just hopefully we don’t get in trouble.’ And … obviously it shows the culture of non-support and definitely not leadership.

They did note a tendency to receive verbally expressed support; however, there was concern that this was not followed through with actions or spending.

Diversity is espoused as important, and it’s Tweeted out, and it’s a goal, but in practical terms, it’s an inconvenience.

It’s become very fashionable to be part of Black Lives Matter and to say, ‘I’m against racism,’ but I haven’t seen that really translate into very much change within our department.

Attendings used less condemning language but also noted a previous culture where racism was a “blind spot” or not frequently discussed or prioritized.

Post-DARE, participants felt physician group-level antiracism awareness had increased. Even focus groups held while DARE was ongoing noted that conversations about equity had become “semi-normalized … people are at least cognizant of the issues, whereas before it was a big blind spot.” Even being “willing to have that conversation…is a huge step forward.” Post-DARE resident groups found that with these increasing conversations and awareness, they felt “safe” and “comfortable” discussing antiracism on shift, because the DARE curriculum made them feel it was “explicit” and “salient.” They described antiracism and equity as “in the waters more,” “in the ether,” “a common footprint” and “a shared understanding.” They found that with this, “the ability to make change is there.” Across training levels, participants noted an increased emphasis on equity work, finding “something that I’ve really enjoyed seeing was that it seems to be at least a priority now.” Some noted a sense of empowerment in their ability to embrace this work:

I have felt more empowered to talk about these issues and also feel like this is okay to have this as a priority, and that this is a focus of being an academic faculty person, whereas I think in the past it was like this is a side interest … I think it’s become more of an interest for other people, and I think that’s been something beneficial.

#### Future Curricular Aims

Post-DARE, participants often described a pull between a changing physician culture and the rest of the ED staff. Most frequently, participants cited a desire for multidisciplinary outreach to all EM staff to incite greater culture change, reporting “I don’t think that we’re going to make big progress on culture until everybody is on the same page and intentional.” There was particular interest in including nurses in antiracism education “given [nurses] are the folks who are at the bedside for the majority of the time with patients.”

Other key areas for future intervention included continuing to have longitudinal learning “sprinkled in throughout the academic year” and incorporating advanced topics that were more intersectional. Participants particularly noted the strong impact that personal stories and narrative had on their understanding. Participants were universally interested in seeing internal care metrics broken down by race as a tool for learning and improvement. A few had concerns about backlash and the need for the appropriate framing of such data.

### Quantitative Results

Fifty-six clinicians (26 attendings, 22 residents, three fellows, and one advanced practice practitioner) were included in the survey data (Table 4).

As DARE sessions were attended variably, it was difficult to estimate the survey response rate. Among total physicians in our trainee and faculty group, this was a survey response rate of 34%, which is an underestimate given that not all faculty attended DARE sessions. The majority of clinicians (48, 85.71%) identified as White, mirroring a majority White faculty and trainee group, and about half were female. The change in retrospective pre/post responses for anti-racism medical practice behaviors for post-intervention survey respondents can be found in Table 5.

Respondents’ assessment of their engagement with anti-racist medical practice behaviors significantly increased from pre- to post-intervention, from an average composite score of 19.27 to 23.23 (*P* < .001). These behaviors included reflecting on how racial bias could impact their practice of medicine, taking steps to mitigate bias in patient care, addressing microaggressions, reporting issues of racism, identifying how structural racism impacts patient care, and voicing concerns or ideas for change around racism to their leadership or community. There were also significant increases in the non-composite items (not included in total score due to not an applicable option for some respondents). Among clinicians who conduct lectures, the integration of health equity, racism, or bias issues into lectures significantly increased from 2.77 to 3.85 (*P* < .001). Among clinicians conducting research, the integration of health equity, racism, or bias issues into their research improved from 3.05 to 3.78 (*P* < .001). Among clinicians in leadership roles, the integration of health equity, racism, or bias issues into their role improved from 3.12 to 3.95 (*P* < .001). Repeated measure analysis of variance findings comparing anti-racism medical practice changes over time by sex and ethnicity can be seen in Appendix B. There were no significant differences over time by sex and ethnicity.

## DISCUSSION

This study demonstrates the potential of an antiracism and equity curriculum to develop an antiracist practice of medicine and encourage an antiracist culture in hospitals. Focus groups revealed the curriculum was overall acceptable to trainees and faculty alike, with some individual sessions favored over others. Opportunities for practicing skills, such as in simulation, were particularly appreciated.

Focus groups suggested that faculty had variable prior exposure to antiracism- or equity-focused education, much of it from individuals seeking it outside their medical training. A subtheme included the actively poor education and hidden curriculum faculty had been presented within their training. This suggests the integral importance of including faculty in antiracism- and equity-focused education. As a participant had reflected after DARE, they were the ones “impacting the upbringing and future careers” of trainees, suggesting the need for them to learn themselves to be better informed leaders and educators. Residents also expressed a need for improved antiracism and equity training, although newer residents reflected they had at least some bias-focused training in medical school. Antiracism and equity training may remain a moving target for trainees as medical schools take on more education in these realms. Regardless, in graduate medical education trainees will continue to need to learn how to apply broad concepts of antiracism and equity to their specific setting. Programs implementing antiracist and equity education should assess the baseline knowledge and skills of their trainees to help inform how much and what type of education is needed.

Assessment of the curriculum in focus groups suggest that it was impactful, further supported by survey responses. Trainees and attendings alike noted a changing knowledge base and overall attitude in the focus groups. Participants noticed differences in how they approached patient interactions, taught learners, and interacted with medical team members. They also noted strides in their abilities to deliver antiracist care while acknowledging the need for further individual growth. This was mirrored in survey questions with significant changes in reflection on and mitigation of personal racial biases, addressing microaggressions, identifying the impact of structural racism on patient care, and proposing changes or bringing up concerns related to local structural racism in care.

A qualitative theme particular to attendings was change to their teaching and leadership after DARE. This was also a significant change in the survey data; for those who had educational, research or leadership positions, they indicated an increase in integration of antiracism and equity in those realms. This does rely on self-report but is bolstered in part by our focus group participants’ focus on culture change: their observation that the colleagues around them were more aware of and invested in antiracism and equity. These findings are encouraging in their suggestion that a curriculum can begin to alter personal *and* group-level attitudes and practices.

For those hoping to initiate an antiracism curriculum, there are now numerous published workshops, simulation scenarios, and lectures that programs can use as building blocks.^23^–^27^ We posit based on our results that the longitudinal, interactive, multimodal, and embedded nature of our curriculum were some of the most important aspects that should be included in future antiracism curricula across the board. We addressed learners at different points in their training and practice, who reported differing, nonlinear educational experiences in this area. This underlines the importance of targeting all levels of learners for antiracism training. Our qualitative themes emphasized the impact of medical culture in learning—whether it be the silent curriculum in medical school learned from clinical rotations, or a faculty member’s perception of what is considered a “priority” by their institution—making medical culture a key target for change.

We would also emphasize the importance of local context, which influenced our approach and certainly should be considered for any institution. For example, our trainees and faculty were both grand majority White, which is why we chose to include a major workshop session focused on privilege and whiteness. Additionally, as confirmed in our initial focus groups, our department was starting from a place without a clear culture or support of antiracism. Our curriculum was initially supposed to include a nursing arm (see Appendix C, Proposal of Nursing DARE Curriculum), to more thoroughly impact departmental culture and practice. However, we ran into such vocal dissent at this idea that it was mandated by the hospital to be put on hold for over a year, and ultimately it fell apart due to lack of support and staff availability. This made clear the underlying objection to antiracism among, at a minimum, a vocal minority of our nursing staff, as well as the antipathy of our hospital. For systems like ours, any curriculum aimed at trainees should also include faculty, and as many other EM/PEM staff as possible, to better encourage a true shift in patient care and allow projects on a systemic scale. As noted in our focus groups, prior to the curriculum residents had faced significant hurdles, as well as fear, around taking an active systemic stance advocating for their patients. After, although we had not yet been able to reach our ideal of including nursing, more trainees and faculty felt that antiracism and racism were concepts that were in the water, and an appropriate focus for a career.

## LIMITATIONS AND NEXT STEPS

We conducted this curriculum and study at a single institution, so this must be considered in its particular context. We were reliant on self-report of knowledge and skills in both our focus group and survey data. While this is common in the assessment of educational interventions,^24^,^28^,^29^ as we look forward to the impact of curricula on patient care and outcomes, more direct measures will be useful in future studies. Notably, our theme of culture change was not an individual self-report but a group reflection on the department as a whole.

Our survey response rate was difficult to calculate and is potentially lower than ideal, opening up the possibility of a positive response bias in the data. Additionally, survey results were partially reliant on retrospective report of attitudes and skills prior to the curriculum. While this was purposeful given the topics taught, there is also potential bias in recall. However, our quantitative data strongly mirrors the themes of our robust qualitative data, suggesting likely validity. In future iterations we would hope to capture a higher proportion of participants via quantitative data.

It is key to note that education is only one arm in improving antiracist care and culture. For example, our focus was not on recruitment and retention, altering hospital policies, or engendering hospital-level culture change. Such interventions are also necessary to fully embrace antiracist medicine at both the individual and institutional level.^30^–^33^ This broader lens will also necessarily begin overlapping with an educational focus. For example, one qualitative subtheme was individual room for improvement in the clinical practice of EM. This will remain an important next step in curriculum development while also requiring creative systemic approaches, such as integrating electronic health dashboards that give racial disparity data or including faculty and trainees in racism-focused quality improvement.^34^–^36^ Finally, a key next step for our institution is providing antiracism education for all team members,^37^,^38^ encouraging a broader culture shift.

## CONCLUSION

Overall, a longitudinal, multimodal antiracism and equity curriculum targeted at both trainees and faculty was effective in creating increased anti-racist attitudes and self-reported behaviors and improved antiracist/equity-focused physician culture. Future directions include expanding the curriculum in new directions, engaging multidisciplinary staff, and targeting internal patient metrics toward equitable care.

## Supplementary Information



## Figures and Tables

**Table 1:** Objectives, framework and assessment map of the Discussing Anti-Racism and Equity curriculum.

Objective	Bloom’s Taxonomy Level	Level of racism	Assessment^a^
Assess implicit bias and the effects of racism on individual practice	Analyze	Personally mediated racism	Survey Item 1–2 + focus group individual practice theme
Identify the legacy and impact of structural racism in medicine	Understand	Institutional racism	Survey Item 5
Use practices that challenge the subtle impacts of individual racism	Apply	Personally mediated racism	Survey Item 3–4 + focus group individual practice theme
Take feasible actions or create goals for an antiracist medical practice	Create	Intuitional, personally mediated, internalized racism	Survey Item 6–9 + focus group individual practice theme
Participate in an actively antiracist department culture	Evaluate	Institution and personally mediated racism	Survey Item 7–9 + focus group culture theme
Develop a core group of participants committed to an anti-racist ethos	Create	Institutional racism	Not formally assessed. Further encouraged with creation of a DEI committee open to new members with a variety of projects, as well as ongoing encouragement of integration of equity topics into education

a Further detail on survey items in Table 5; further detail on focus group themes in Table 3.

*DARE*, Discussing Anti-Racism and Equity; *DEI*, diversity, equity, and inclusion.

**Table 2:** Sessions of the Discussing Anti-Racism and Equity curriculum (DARE),

Lecture/ workshop session	Topics covered
PEM/PICU joint conference: Who Me? Bringing awareness of racial bias and an anti-racist ethic to our everyday practice.	Implicit bias, impact of racist implicit bias on patient care and communication, introduction to microaggressions with small-group discussion, upstander skills review and practice/role play
Discussing Antiracism and Equity: Breaking our Allegiance to Whiteness (guest speaker)	Introduction to privilege, conceptions of White identity, small-group discussion around personal racial identity
Antiracism Policies and Practices for our Organization	Institutional policies that relate to antiracism, local related research work, educational impacts, mutual accountability and collective responsibility
Grand Rounds: Injury, Equity and Racism (guest speaker)	Injury prevention, inequities in injury burden, identifying inequity causes and solutions
Microaggressions and Implicit Bias Case-Based Workshop	Introduction to microaggressions, upstander skills review, small-group case review and practice/role play
Systemic Racism and Health (guest speaker)	Discussion of systemic racism, impact of structural factors on medicine and health, opportunities for collective action
Racism-focused M&M	Case evaluation with racism focus
Race-based Research	Race-based biases in clinical research, practical strategies to identify and mitigate bias in research, intersectionality
PEM: Use of race in pediatric algorithms	Discussion of use of race in pediatric algorithms, specific example--debate of UTI algorithm
Medical Apartheid: History of racism in medical research and impact on current practices	Racism in medical research throughout US history, including voices of dissent; impact on informed consent and current emergency medicine practices; influence of historical abuses on current practices and patient perspectives
Institutional Antiracism Updates	Intermittent (roughly biannual) brief updates reviewing departmental and institutional antiracism actions
EM Intern Orientation: Introduction to Racism and Microaggressions^a^	Racism and levels of racism, upstander skills and practice
Simulation Cases	
Interrupting Overt Racism in the Workplace	Addressing overt racism, options when patients make racist statements, protective institutional policies and resources
Interprofessional Microaggressions	Recognizing and interrupting interprofessional microaggressions, microaggressions where power differentials are in play
Implicit Bias and Patient Care: Mitigating Bias, Preventing Harms	Identifying interactions where racial implicit bias is impacting patient care, practicing taking action, racism in child abuse evaluation
Movies	
Juneteenth discussion: Movie screening and discussion of the Netflix movie 13th	
A Baldwin Kind of Mood: Movie screening and discussion of interview with James Baldwin	
Book Club	
How to Be an Antiracist	
Talking to Strangers	
Caste	
White Fragility	
Medical Apartheid	

a Implemented for new interns after the prior academic year of the DARE curriculum had run.

*M&M*, morbidity and mortality; *PEM*, pediatric emergency medicine; *PICU*, pediatric intensive care unit; *UTI*, urinary tract infection.

**Table 3:** Focus group themes.

Themes	Sub-themes
Non-standardized prior education	Limited/inadequate prior learning
	Influence of silent curriculum
	Self-directed learning
Impact of DARE on individual attitudes and practice	Increased bias awareness (self and others)
	Specific knowledge points or skills
	Eagerness to brainstorm new directions and push the department as a whole
	Room for improvement in individual practices
Departmental culture before and after DARE	Pre-DARE antiracism resistance or blind spot
	Increased group-level awareness after DARE
	Perceived acceptance of antiracism initiatives, empowerment post-DARE
	Departmental or hospital-level disconnect from anti-racism awareness
Future desires	Ongoing curriculum with new topics
	Strong impact of personal stories and narratives
	Equity-focused patient care metrics
	Multidisciplinary outreach to all EM staff

*DARE*, Discussing Anti-Racism and Equity; *EM*, emergency medicine.

**Table 4:** Demographics of survey respondents.

	n (%) (N=56)
Gender	
Male	27 (48.21%)
Female	29 (51.79%)
Work Role	
Physician	55 (98.21%)
Advanced practice practitioner	1 (1.79%)
Physician role^a^	
Attending	26 (47.27%)
Fellow	3 (5.45%)
Resident	22 (40.00%)
Attending who completed residency in 2021	4 (7.27%)
Race	
White	48 (85.71%)
Asian	4 (7.14%)
Black	2 (3.57%)
Hispanic/Latinx	2 (3.57%)
Post-training	
0–5 years	5 (19.23%)
6–10 years	4 (15.38%)
11–15 years	4 (15.38%)
16–20 years	6 (23.08%)
21–30 years	6 (23.08%)
>30 years	1 (3.85%)

a Frequencies are among clinicians who indicate “physician” as their work role.

**Table 5:** Retrospective anti-racism medical practice score (N=56).

	Pre mean (SD)	Post mean (SD)	t (df) *P*-value
Overall score	19.27 (5.57)	23.23 (3.54)	t (55) = − 6.28, P < .001
Item 1. I reflect on how my racial bias might impact my practice of medicine.	3.86 (0.98)	4.39 (0.62)	t (55) = −3.98, P < .001
Item 2. I take steps to mitigate my own racial biases in patient care.	3.66 (1.10)	4.36 (0.59)	t (55) = −5.08, P < .001
Item 3. I address microaggressions or biased statements with a colleague or patient.	3.11 (1.12)	3.57 (0.97)	t (55) = −3.33, P < .01
Item 4. I report issues of individual racism when unable to confront it myself.	2.82 (1.10)	3.46 (1.04)	t (55) = −5.00, P < .001
Item 5. I learn and identify how structural racism may impact patient care.	3.18 (1.22)	4.18 (0.81)	t (55) = −6.63, P < .001
Item 6. I propose changes or bring up concerns to improve structural racism in my hospital/department/community.	2.64 (1.18)	3.27 (1.12)	t (55) = −4.85, P < .001
Additional items on the integration of health equity, racism and/or bias			
Item 7. I integrate issues of health equity, racism, or bias into lectures that I give.	2.77 (1.24)	3.85 (1.14)	t (45) = −6.01, P < .001
Item 8. I integrate issues of health equity, racism, or bias into the research that I do.	3.05 (1.39)	3.78 (1.29)	t (32) = −4.42, P < .001
Item 9. I integrate issues of health equity, racism, or bias into the leadership position I hold.	3.12 (1.14)	3.95 (1.13)	t (39) = − 5.24, P < .001
