# Supplementary material for: Moving Beyond “Check A Box”: Shifting Physician Perceptions and Culture with an Antiracism and Equity Curriculum
Source: West J Emerg Med. 2025 May 19;26(3):441–51. doi: 10.5811/westjem.20797 (PMC12208040; doi:10.5811/westjem.20797)
Supplement: Supplementary file 1 [file wjem-26-441-s001.docx]

**Appendix A.** Anti-Racism Medical Practice Survey Tool.

Please select the answer choice that best reflects your **current** practice or thoughts regarding the following items:

|  | I am not interested in this practice | I would like to do this but have not considered how | I have been thinking about how to do this | I sometimes do this | I often or always do this |
| --- | --- | --- | --- | --- | --- |
| I reflect on how my racial bias might impact my practice of medicine |  |  |  |  |  |
| I take steps to mitigate my own racial biases in patient care |  |  |  |  |  |
| I address microaggressions or biased statements with a colleague or patient |  |  |  |  |  |
| I report issues of individual racism when unable to confront it myself |  |  |  |  |  |
| I learn and identify how structural racism (department, hospital level or beyond) may impact patient care |  |  |  |  |  |
| I propose changes or bring up concerns to improve structural racism in my hospital/department/community |  |  |  |  |  |

Please select the answer choice that best reflects your **current** practice or thoughts regarding the following items:

|  | I am not interested in this practice | I would like to do this but have not considered how | I have been thinking about how to do this | I sometimes do this | I often or always do this | N/A |
| --- | --- | --- | --- | --- | --- | --- |
| I integrate issues of health equity, racism, or bias into lectures that I give |  |  |  |  |  |  |
| I integrate issues of health equity, racism or bias into the research that I do |  |  |  |  |  |  |
| I integrate issues of health equity, racism or bias into the leadership position I hold |  |  |  |  |  |  |

Please select the answer choice that best reflects your practice or thoughts **prior to fall 2020**,

(prior to DARE curriculum):

|  | I was not interested in this practice | I would have liked to do this but had not considered how | I had been thinking about how to do this | I sometimes did this | I often or always did this |
| --- | --- | --- | --- | --- | --- |
| I reflected on how my racial bias might impact my practice of medicine |  |  |  |  |  |
| I took steps to mitigate my own racial biases in patient care |  |  |  |  |  |
| I addressed microaggression or biased statements with a colleague or patient |  |  |  |  |  |
| I reported issues of individual racism when unable to confront it myself |  |  |  |  |  |
| I learned and identified how structural racism (department, hospital level or beyond) may impact patient care |  |  |  |  |  |
| I proposed changes or brought up concerns to improve structural racism in my hospital/department/community |  |  |  |  |  |

Please select the answer choice that best reflects your practice or thoughts **prior to fall 2020**,

(prior to DARE curriculum):

|  | I was not interested in this practice | I would have liked to do this but had not considered how | I had been thinking about how to do this | I sometimes did this | I often or always did this | N/A |
| --- | --- | --- | --- | --- | --- | --- |
| I integrated issues of health equity, racism, or bias into lectures that I gave |  |  |  |  |  |  |
| I integrated issues of health equity, racism or bias into the research that I did |  |  |  |  |  |  |
| I integrated issues of health equity, racism or bias into the leadership position I held |  |  |  |  |  |  |

**Appendix B**. Comparing anti-racism medical practice composite scores by gender and ethnicity (n=56).

|  | Pre-Score | Post-Score | F (df), *P* |
| --- | --- | --- | --- |
|  | mean (SD) | mean (SD) |  |
| Gender |  |  | F (1) = 0.0, *P* = .99 |
| Male (n=27) | 18.74 (5.04) | 22.70 (3.67) |  |
| Female (n=29) | 19.76 (6.07) | 23.72 (3.41) |  |
| Ethnicity |  |  | F (1) = 0.29, *P* = .59 |
| Non-White (n=8) | 17.25 (5.26) | 22.38 (3.16) |  |
| White (n=48) | 19.60 (5.60) | 23.38 (3.61) |  |

*df*, degree of freedom**.**

**Appendix C.** Proposal for nursing DARE* curriculum.

| **Proposal: Health Equity, Implicit Bias and Anti-Racism – Improving Care for our Emergency Department Patients** |
| --- |
| This series of virtual interactive sessions for nursing staff is proposed to give an introduction to implicit bias and racism as it affects patient care. These sessions would be required, lasting about 60 minutes each total, over Zoom. A number of available dates for each session would be posted for people to sign up for. We will work with nursing leadership to ensure these sessions are either monetarily or time reimbursed. We plan to apply for sessions to count as CNE. This curriculum is being built by a multidisciplinary group of emergency physicians and nurses with either experience in or dedication to antiracism and equity education. Emergency physicians and APPs will be receiving a parallel curriculum concurrently. |
| *In brief, these 3 sessions would include:* |
| Session #1: Introduction to terminology and implicit bias |
| - Review definitions of implicit bias, racism and anti-racism. |
| - Discuss case examples of how racial bias can impact patient care. |
| Session #2: Overview of historical and structural racism in medicine |
| - Review key illustrative examples of medical racism in history. |
| - Review how structural racism continues to affect health and the practice of medicine, using case examples. |
| - Discuss implications for care of patients in our ED. |
| Session #3: Microaggressions |
| - Define microaggressions |
| - Brainstorm and review examples |
| - Introduce tools for addressing microaggressions in the ED |
| - Practice using these tools |

**DARE*, Discussing Anti-Racism and Equity ; *APP*, advanced practice practitioner; *ED*, emergency department.
